# An *in vitro* reconstituted U1 snRNP allows the study of the disordered regions of the particle and the interactions with proteins and ligands

**DOI:** 10.1093/nar/gkab135

**Published:** 2021-03-02

**Authors:** Sébastien Campagne, Tebbe de Vries, Florian Malard, Pavel Afanasyev, Georg Dorn, Emil Dedic, Joachim Kohlbrecher, Daniel Boehringer, Antoine Cléry, Frédéric H-T Allain

**Affiliations:** Institute of Biochemistry, Department of Biology, ETH Zurich, Hönggerbergring 64, CH-8093 Zürich, Switzerland; Institute of Biochemistry, Department of Biology, ETH Zurich, Hönggerbergring 64, CH-8093 Zürich, Switzerland; Institute of Biochemistry, Department of Biology, ETH Zurich, Hönggerbergring 64, CH-8093 Zürich, Switzerland; Cryo-EM Knowledge Hub (CEMK), ETH Zurich, Hönggerbergring 64, CH-8093 Zürich, Switzerland; Institute of Biochemistry, Department of Biology, ETH Zurich, Hönggerbergring 64, CH-8093 Zürich, Switzerland; Institute of Biochemistry, Department of Biology, ETH Zurich, Hönggerbergring 64, CH-8093 Zürich, Switzerland; Paul Scherrer Institute, SINQ facility, Villigen, Switzerland; Cryo-EM Knowledge Hub (CEMK), ETH Zurich, Hönggerbergring 64, CH-8093 Zürich, Switzerland; Institute of Biochemistry, Department of Biology, ETH Zurich, Hönggerbergring 64, CH-8093 Zürich, Switzerland; Institute of Biochemistry, Department of Biology, ETH Zurich, Hönggerbergring 64, CH-8093 Zürich, Switzerland

## Abstract

U1 small nuclear ribonucleoparticle (U1 snRNP) plays a central role during RNA processing. Previous structures of U1 snRNP revealed how the ribonucleoparticle is organized and recognizes the pre-mRNA substrate at the exon–intron junction. As with many other ribonucleoparticles involved in RNA metabolism, U1 snRNP contains extensions made of low complexity sequences. Here, we developed a protocol to reconstitute U1 snRNP *in vitro* using mostly full-length components in order to perform liquid-state NMR spectroscopy. The accuracy of the reconstitution was validated by probing the shape and structure of the particle by SANS and cryo-EM. Using an NMR spectroscopy-based approach, we probed, for the first time, the U1 snRNP tails at atomic detail and our results confirm their high degree of flexibility. We also monitored the labile interaction between the splicing factor PTBP1 and U1 snRNP and validated the U1 snRNA stem loop 4 as a binding site for the splicing regulator on the ribonucleoparticle. Altogether, we developed a method to probe the intrinsically disordered regions of U1 snRNP and map the interactions controlling splicing regulation. This approach could be used to get insights into the molecular mechanisms of alternative splicing and screen for potential RNA therapeutics.

## INTRODUCTION

RNA processing is an essential part of eukaryotic gene expression that consists of triggering coordinated RNA modifications required for the maturation of the pre-messenger RNA into the mRNA. As soon as the nascent RNA emerges from the RNA polymerase II (RNAP) exit channel, the capping enzymes ensure the addition of the cap at the 5′-end, the spliceosome removes the intervening sequences and the 3′-end processing machinery cleaves and polyadenylates the transcript. All these steps are coordinated by the RNAP C-terminal domain post-transcriptional modifications that recruit most of the RNA processing machineries on this landing pad during transcription (1). Among those, U1 small ribonucleoparticle (U1 snRNP) plays a central role in RNA processing and controls RNA splicing (2), transcription efficiency and 3′-end processing (3).

During the assembly of the major spliceosome, U1 snRNP binds to and defines the 5′-splice site (5′-ss) co-transcriptionally (4,5). When the sequence of the 5′-ss is not optimal or sequestered in secondary structures, splicing regulation is prone to occur. About 95% of human genes have alternatively spliced variants and this mechanism strongly contributes to the high metazoan protein diversity and gene expression adjustments (6). In these cases, *trans* splicing factors specifically recognize *cis* RNA elements at the surroundings of the weak splice site and modulate the recruitment of the splicing machinery including U1 snRNP (7). Beside its role in the nucleation of the spliceosome, U1 snRNP regulates chromatin retention of long non-coding RNAs (8) and controls 3′-end processing through the mechanism of U1 telescripting (9,10). Due to its major role in RNA processing, perturbations in U1 snRNP homeostasis or deleterious mutations modifying its function were associated with genetic diseases, including neurodegenerative diseases (11,12) and cancers (13,14). Recent advances in the studies of U1 snRNP activity modulation by artificial splicing effectors have highlighted potential therapeutic applications (15–17).

Mammalian U1 snRNP is composed of the 164 nucleotides-long U1 snRNA, seven Sm proteins (SmB/SmB′, SmD1, SmD2, SmD3, SmE, SmF and SmG) and three U1-specific proteins (U1–70K, U1-A and U1-C). Previous structures of U1 snRNP (18–20) revealed that the RNA adopts a trefoil fold on which the heptameric Sm core assembles between stem loops 3 and 4. The stem loops 1 and 2 are specifically recognized by U1–70K and U1-A N-terminal RNA Recognition Motifs (RRM), respectively. A striking feature of the structure is the N-terminal region of U1–70K, which wraps around the Sm ring and enhances the binding of U1-C. The RNA duplex formed upon 5′-ss recognition is further stabilized by the U1-C zinc finger that binds the minor groove of the intermolecular RNA helix at the exon-intron junction. Mammalian and yeast pre-spliceosome structures (21,22) revealed that other proteins (as LUC-7 in yeast and Prp28 in human) could help stabilize the U1 snRNP/5′-ss interface. Besides the well-defined structure of the particle core, almost 40% of the protein components (i.e. C-terminal extensions of U1–70K, U1-A, U1-C, Sm B/B’, Sm D_1_ and Sm D_3_) are made of low complexity sequences and repeated motifs that were not observed in previous structures. Their functional importance in spliceosome assembly (21,23), U1 snRNP biogenesis (24) and alternative splicing regulation (25) was previously reported. Some of these extensions, such as the tails of Sm B/B’, Sm D_1_ or the long RS domain of U1–70K, are specific to the mammalian spliceosome and are not found in the yeast U1 snRNP. Finding a way to probe the U1 snRNP tails at atomic detail still represent a challenge for structural biology.

The initial steps of spliceosome assembly are transient and prone to regulation (26). They determine the splicing fate. The reaction starts with the recognition of the 5′-ss that directly base pairs with the 5′-end of U1 snRNA. Even if the 5′-end of U1 snRNA can accommodate degenerated 5′-ss, the presence of bulged nucleotides or shifted base pairing was associated with alternative splicing and mutations inducing various diseases (27–29). Notably, we have recently shown in the study of *SMN2* splicing modifiers that it was possible to study interactions between U1 snRNP-5′-ss complexes and small molecule effectors using NMR spectroscopy (17). The small molecule represents the minimal splicing factor since it creates a link between the pre-mRNA and U1 snRNP and therefore stabilizes U1 snRNP on the weak 5′-ss of *SMN2* exon 7. Proteic splicing factors also communicate with U1 snRNP in order to modulate its recruitment on weak 5′-ss. The U1 snRNP tails and more precisely the RS domain of U1–70K was proposed to drive interactions with SR proteins through protein-protein interactions (30–32). U1 snRNP also contains two stem loop structures that are free of U1-specific proteins and exposed at the surface of the particle. The stem loops 3 and 4 are responsible for interactions with the splicing regulators FUS (33) and PTBP1 (34), respectively. Polypyrimidine tract binding protein 1 (PTBP1) is a well-studied splicing factor that promotes or represses spliceosome assembly depending on the position of the *cis* RNA element with respect to the splice site (35). The stem loop 4 is essential for splicing (36) and recently, the U2 snRNP component SF3A1 was shown to establish direct contacts with U1 snRNA stem loop 4 during spliceosome assembly (37). PTBP1 is composed of four RRM domains that all target pyrimidine rich sequences (38). The RRM domains of PTBP1 have similar sequence specificities: RRM1–4 recognizes YCU, CU(N)N, YCUNN and YCN, respectively (Y indicating a pyrimidine and N any nucleotide). Although RRM1 and RRM2 tumble independently in solution, RRM3 and RRM4 interact together to form an independent module promoting RNA looping (39,40). PTBP1 induces splicing regulation by contacting U1 snRNP through the terminal stem loop 4 of the U1 snRNA (34). However, it remains unclear whether the splicing factor contacts U1 snRNP protein components as well. NMR spectroscopy is potentially a straightforward technique to get structural insights into the labile interactions between the splicing factors and the entire U1 snRNP.

Here, we developed a method to produce U1 snRNP using mostly full-length components in order to observe the core as well as the tails of the ribonucleoparticle (RNP) using NMR spectroscopy. Our results revealed that the tails are intrinsically disordered regions in the context of the particle except for the C-terminal RNA recognition domain of U1-A. The interaction between U1 snRNP and the splicing factor PTBP1 was also monitored by NMR spectroscopy. Altogether, the approach described below sheds light on the flexible parts of U1 snRNP and opens the door to structural studies of alternative splicing regulation in solution.

## MATERIALS AND METHODS

### Cloning, expression and purification of proteins

The procedures of cloning, expression and purification are described in the supplementary materials.

### Preparation of U1 snRNA

The U1 snRNA was transcribed *in vitro* using homemade T7 RNA polymerase with the plasmid pUC19-U1 snRNA previously linearized by SalI. pUC19-U1 snRNA contains a T7 promoter followed by the hammerhead ribozyme in fusion with the sequence coding for the U1 snRNA and a SalI cleavage site. The transcription mixture was applied to a high-performance liquid chromatography (HPLC) system that separates RNAs on an anion exchange column at 85°C and in denaturing conditions (6 M Urea). Fractions containing the U1 snRNA were precipitated using butanol and dissolved in water (three times) to remove residual urea. The RNA was refolded, lyophilized and stored at −20°C. The same procedure was followed to prepare U1 stem loop 2 (5′-GGGAUCCAUUGCACUCCGGAUCCC-3′) and 4 (5′-GGGACUGCGUUCGCGCUUUCCC-3′) from oligonucleotide templates. The 5′-ss oligonucleotide (5′-GGGUAAGUCU-3′) was purchased (Dharmacon).

### Reconstitution of U1 snRNP

About 6 nmoles of each of the Sm protein heterodimers were mixed together in 500 μl buffer A (Hepes 10 mM pH7.5, KCl 250 mM, DTT 5 mM) and 4 nmoles of the U1 snRNA was added. The mixture was incubated at 30°C for 30 min and 15 min at 37°C. Then, the mixture was incubated in ice and 1 equimolar amount (4 nmoles) of U1–70K was added. Fifteen minutes later, one molar equivalent (4 nmoles) of U1-A was added and the mixture was incubated during 12 h in ice. U1 snRNPΔU1-C was purified by anion exchange using a 1 ml MonoQ column (Pharmacia) previously equilibrated in buffer A. The sample was loaded on the column and eluted with a gradient of KCl (buffer B: Hepes 10 mM pH 7.5, KCl 2M, DTT 5 mM). The complex elutes at roughly 25% of buffer B. U1 snRNPΔU1-C was then dialyzed against buffer C (sodium phosphate 10 mM pH6.8, NaCl 100 mM, DTT 5 mM). The concentration of U1 snRNPΔU1-C was determined and 1.5 molar equivalent of U1-C was added. The mixture was concentrated to 250 μl by centrifugation and separated by size exclusion chromatography using a Superdex 200 increase in buffer C. Note that for the preparation of some of the NMR samples, the anion exchange step was skipped after verifying on a small fraction of the sample that the free U1 snRNA and misassembled complexes were negligible after reconstitution.

### Small angle neutron scattering

Small angle neutron scattering experiments were recorded at the SANS-I and SANS-II facilities, Swiss Spallation Neutron Source, SINQ, Paul Scherrer Institute, Switzerland. Scattering density matching points for RNA and protein with respect to the D_2_O/H_2_O ratio of the buffer (10 mM NaPO4, pH 6.8, 100 mM NaCl, 2 mM DTT) were determined by contrast variation and extrapolation of *I*_0_ to be 48% D_2_O for protonated components (RNA and protein), and 42% or 100% for protonated or deuterated protein, respectively. Free protein and RNA were recorded at a wavelength of the neutron beam of 6 Å, a collimation of 6 m and a detector distance of 2 and 6 m. Reconstituted U1 snRNP containing ^2^H-U1-A (deuterated at ∼80%) were dialysed for 24 h against suitable buffers. Each reference cuvette was filled with the corresponding dialysis buffer. Complexes were measured at SANS-I at a wavelength of 4.5 Å at two detector distances (2 and 6 m). Scattered neutrons were detected using a 2D 96 cm x 96 cm detector with a pixel size of 0.75 cm. Reduction and analysis of SANS data were performed with the program BerSans (41) and visualized using Primus QT from the ATSAS package (42). Beamline specific correction factors for data reduction are 1.338 and 1.284 for data recorded at a wavelength of 4.5 and 6 Å, respectively. After reduction, data were confronted to back calculated curves generated with CRYOSON (42) based on the available crystal structure of U1 snRNP (18).

### Sample preparation for electron microscopy

About 200 pmoles of freshly purified U1 snRNP was submitted to zonal centrifugation on a 10–30% glycerol gradient containing 0.025% glutaraldehyde (43). The centrifugation was performed at 4°C using the SW-41Ti rotor during 19 h at 39 000 rpm. Density gradients were fractionated using the Foxy Jr. system in 500 μl fractions containing 50 μl of 1 M Tris pH 7.5 in order to quench the glutaraldehyde. The fraction containing the complex of interest was washed 7 times with buffer D (Hepes 10 mM pH7.2, KCl 50 mM, DTT 5 mM) and diluted to 70 nM in presence of 0.002% NP-40. For the sample characterization by the negative staining, fresh U1 snRNP sample (3 μl) was applied on glow-discharged Quantifoil Carbon supported grids (Cu 300 mesh) for 1 min, washed twice with water and stained for 1 min in 1% uranyl acetate. For cryo-EM grid preparation, fresh U1 snRNP sample (3 μl) was applied on glow-discharged Quantifoil (R2/2, Cu 300-mesh) grids with an ultrathin (∼2 nm) carbon coating. The specimen was plunge frozen with a Mark IV Vitrobot (Thermo Fisher Scientific) in a mixture of ethane and propane cooled by the liquid nitrogen with the following settings: 2–3 s blotting time; blot force 0; 15°C and 100% humidity.

### Cryo-EM data collection and processing

The cryo-EM dataset was collected using a Titan Krios microscope (Cs = 2.7 mm) operated at 300 keV, equipped with a Gatan K3 detector with GIF-quantum energy filter (20 eV slit width). About 5155 exposures were collected in a dose-fractionation mode with 30 frames per exposure, at a nominal magnification of 105 000× (corresponding to a calibrated pixel size of 0.84 Å/pix), with the total dose over the exposure time of 1.85 s was ∼80 e-/Å^2^. Fully automated data collection was carried out using the EPU software (Thermo Fisher Scientific) with a target defocus range from -1.8 to -3 μm. The collected dataset was subjected to motion-correction and dose-weighting with MotionCor2 (44), followed by CTF-determination by Gctf 1.06 (45) and particle picking using CRYOLO software (46). About 1.4 million particles were picked followed by standard routines of 2D-classifications in Relion 3.1 (47). The results of the classification are presented in Figure 2B. Randomly chosen subset of 100 000 particles was used for obtaining a reliable *ab initio* 3D-reconstruction in cisTEM software (48). Further 3D-analysis revealed high level of conformational heterogeneity of the dataset. A number of rounds of subsequent 3D-classifications using various parameters and masks were attempted to separate out a single solid class, which revealed the best one composed of ∼14 000 particles. The subset of particles was further refined to 9.8 Å using SIDESPLITTER software (49) within the Relion package. Atomic model of U1 snRNP (17) was then fitted in the resulting derived volume using USCF Chimera (50).

### NMR spectroscopy and structure calculation

All the NMR measurements were performed at 313 K using cryo-probed AV IIIHD 600 MHz, AV IIIHD 700 Mhz or AV IIHD 900 Mhz NMR spectrometers (Bruker). Data were processed using Topspin 3.1 (Bruker) and analysed with CARA (51). NMR fingerprints were collected using the 2D ^15^N-^1^H TROSY HSQC or 2D ^13^C-^1^H HMQC experiments. Backbone resonances assignments of U1-A RRM2 and U1-A RRM2-linker were performed using the classical approach by combining triple resonance experiments (3D HNCACB, 3D CBCA(CO)NH and 3D HNCO). Automatic backbone assignment was performed using CARA and AutoLink4 (52). Backbone chemical shifts were used as input to predict secondary structures using TALOS+ and to generate dihedral restraints for structure calculations (53). For U1-A RRM2-linker, side chain assignment was performed by analyzing TOCSY experiments (3D H(CCO)NH, 3D (H)C(CO)NH and 3D HCCH-TOCSY) and NOE derived distances were extracted from 3D ^15^N-^1^H HSQC NOESY, 3D ^13^C_ali_-^1^H HSQC NOESY and 3D ^13^C_aro_-^1^H HSQC NOESY recorded with 80ms mixing time. Chemical shifts and NOESY spectra were used as input for automatic peak picking, NOE assignment and structure calculation with the ATNOS/CANDID/CYANA suite (54) followed by automated assignments within the NOEASSIGN module of CYANA 3.0 (55). The structures were refined in the Cartesian space using the SANDER approach of AMBER20 (56). Analysis of refined structures was performed using PROCHECK-NMR (57). ^15^N relaxation experiments were recorded using classical pulse programs (58). Briefly, the ^15^N longitudinal relaxation rates were extracted from inversion recovery-based experiments using delays varying between 10 ms to 2 s. ^15^N transverse relaxation rates were extracted from Carr–Purcell–Meiboom–Gill based experiments using delays varying between 20 and 250 ms. Data were integrated with CARA and exponential decays were fitted with GraphPad.

## RESULTS

### *In vitro* reconstitution of U1 snRNP

**Figure 1.**
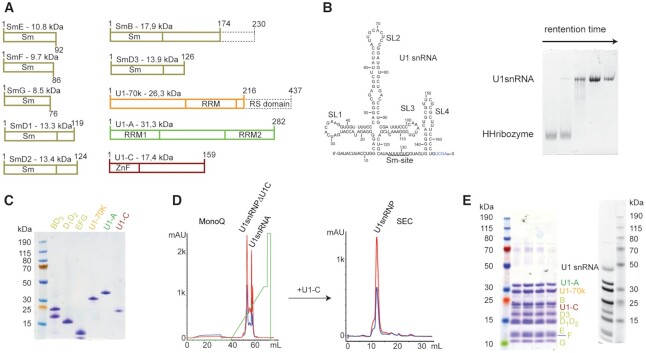
Reconstitution of U1 snRNP. (**A**) Scheme of the U1 snRNP protein components. Dashes boxes correspond to the parts that were not included in our constructs. (**B**) Scheme of the U1 snRNA used for this study. On the right, a 10%-acrylamide urea PAGE stained with toluidine illustrates the separation between the hammerhead ribozyme and the U1 snRNA using purification in denaturing conditions. (**C**) SDS-PAGE stained with Coomassie showing the purified U1 snRNP protein components. (**D**) Purification of U1 snRNP. MonoQ was used as the anion exchange chromatography column and SEC stands for size exclusion chromatography. (**E**) SDS-PAGE gel stained with Coomassie (left) or with silver nitrate (right) showing the purified U1 snRNP.

In order to reconstitute U1 snRNP *in vitro*, the protein components were produced in bacteria and the U1 snRNA was generated by *in vitro* transcription (Figure 1A and B). As previously done (18,20,59–61), the seven Sm proteins were produced as heterodimers or trimer (Sm B/B’-D_3_, Sm D_1_-D_2_ and Sm E-F-G). The three U1 specific proteins U1–70KΔRS, U1-A (or U1-A RRM1 1–117) and U1-C (or U1-C zinc finger 1–61) were expressed individually. Altogether, six bacterial cultures were required to produce the full-length protein components of U1 snRNP except for Sm B/B’ that was cut at amino acid 174 and U1–70K that lacks its RS domain (1–216). The purification of the U1 snRNP protein components is relatively tedious since it requires 13 steps of chromatography and takes about 2–3 weeks (Figure 1C and Supplementary Figure S1). However, large-scale protein stocks can be stored at −80°C. The production of the U1 snRNA was performed by T7-based *in vitro* transcription using a plasmid encoding for a hammerhead ribozyme followed by the U1 snRNA sequence (Supplementary Figure S2). The U1 snRNA produced in this study lacks the pseudo uridines at the 5′-end and the modified cap. Thus, the reconstituted U1 snRNP may have a slightly different 5′-splice site specificity (62) and an altered splicing activity (63). However, we have previously shown that the particle binds to 5′-splice sites and pre-mRNA fragments (17).

The particle was assembled in absence of chaperones and processing enzymes in a stepwise manner that was proposed to occur in the cell (64), even if the timing for U1-A binding to the U1 snRNA is still controversial (65,66). First, we assembled the Sm core on the U1 snRNA and the pre-particles were stabilized by the addition of U1–70K. U1-A was further added and the mixture was incubated on ice overnight. U1 snRNPΔU1-C was then purified by anion exchange chromatography in order to remove the misassembled particles as well as the free U1 snRNA (Figure 1D). The sample was dialyzed to reduce the amount of salt and subsequently U1-C and the 5′-ss were added. The particles were further purified by size exclusion chromatography (Figure 1D). The quality of the reconstitution was assessed using gel electrophoresis (Figure 1E) and CLIR-MS/MS (67). This protocol allowed the production of large amounts of U1 snRNP (up to 50 nmoles) compatible with structural biology approaches including NMR spectroscopy. By producing all the U1 snRNP components individually, this approach offers all possible isotope-labeling schemes and allows the observation of one or several proteins at a time.

### Biophysical validation of the assembly

**Figure 2.**
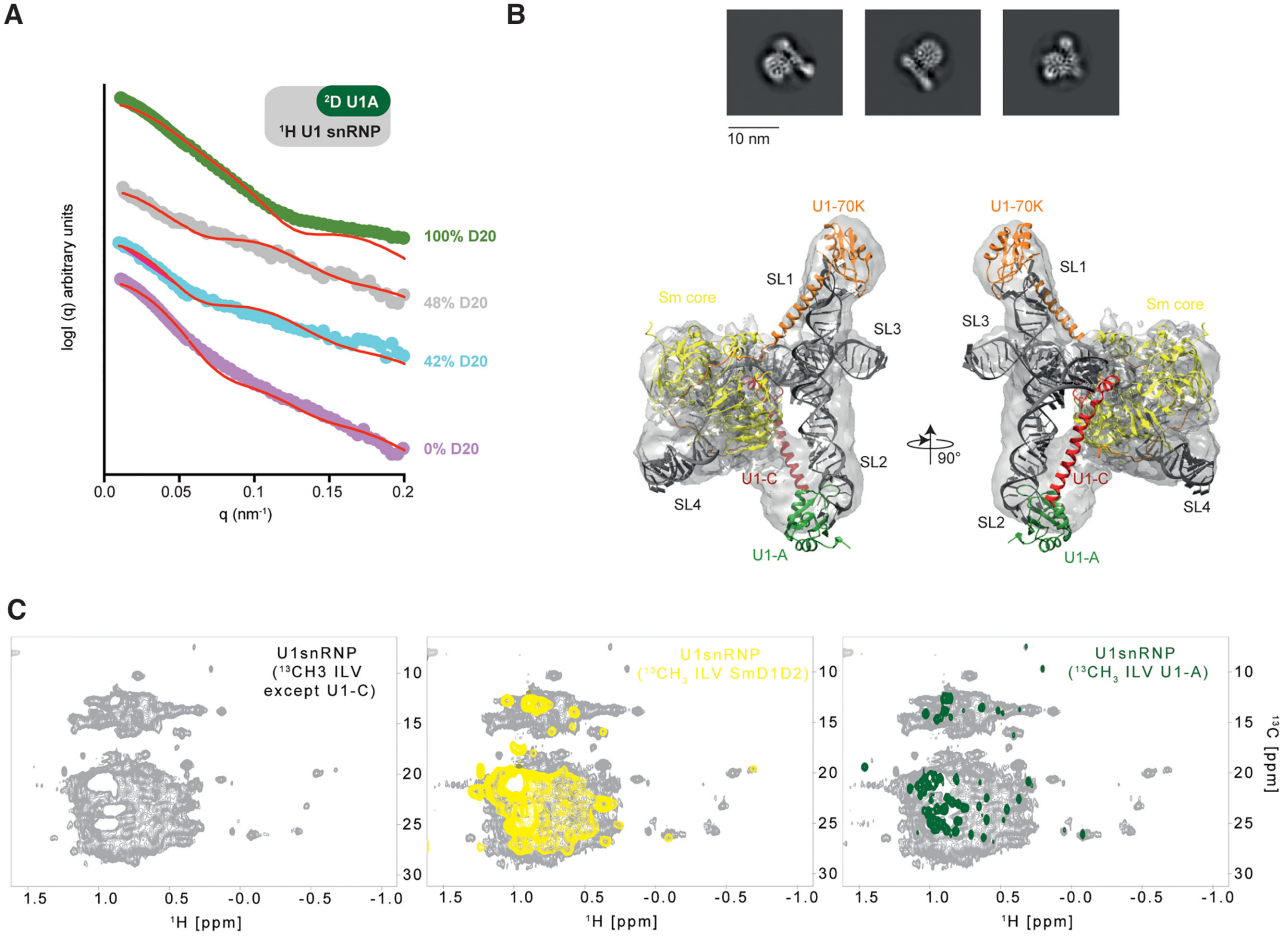
Structural analysis of the *in vitro* reconstituted U1 snRNP. (**A**) Plot of the scattered intensities as a function of the *q* factor (or angle). Experimental curves are colored according to the percentage of D_2_O included in the solvent (0, 42, 48 and 100% are shown in pink, blue, gray and green, respectively). Back calculated curves are shown as red curves. The following χ2 2.72, 1.89, 1.98 and 1.82 were obtained for the data recorded in 100, 48, 42 and 0% D_2_O. (**B**) Representative 2D class-averages of the U1 snRNP particles. Atomic model of U1 snRNP (17) rigid-body fitted into the 9.8 Å resolution cryo-EM map. (**C**) NMR spectra of U1 snRNP. On the left, 2D ^1^H-^13^C HMQC of U1 snRNP ^13^C-labeled on ILV methyl groups of all the proteins except U1-C. In the middle, the yellow spectrum corresponds to the 2D ^1^H-^13^C HMQC of U1 snRNP ^13^C-labeled on ILV methyl groups of Sm D_1_-D_2_ in U1 snRNP. On the right, the green spectrum corresponds to the 2D ^1^H-^13^C HMQC of U1 snRNP ^13^C-labeled on ILV methyl groups of Sm D_1_-D_2_ in U1 snRNP.

In order to validate our reconstitution protocol, we first investigated the overall shape of the purified particles using small angle neutron scattering (SANS) experiments and the contrast matching approach (68,69). By including ^2^H-labeled U1-A in U1 snRNP, neutron scattering curves were collected at different ratio H_2_O:D_2_O to bleach the signal of the different components. In 100% H_2_O, all the components of the particle contribute to the scattered signal. Using this curve, we could determine that the particle has a radius of gyration (*R*g) of 5.06 ± 0.18 nm, an estimated molecular weight of 220 kDa and a maximal distance of 19 nm. All these constants are in agreement with the expected dimensions of the *in vitro* reconstituted U1 snRNP described in this study. At 42%, 48% and 100% D_2_O, the contributions to the scattering of the protonated proteins, the protonated proteins and RNAs and the deuterated protein were bleached, respectively. The experimental data were then compared to simulated curves generated with the crystal structure of U1 snRNP that does not include the C-terminal parts of U1-A, U1-C and Sm proteins (Figure 2A). At low *q* ranges, both curves, the experimental and the simulated ones, matched well, in line with a very similar general shape and dimensions. However, at higher *q* ranges, the curves started to diverge. Scattering at high *q* ranges contains information on details and these differences might be explained by the presence of additional tails in the particles we reconstituted. Overall, the SANS data showed that the *in vitro* reconstituted U1 snRNP has the expected shape and confirmed the additional elements that are not in the crystal structure.

To get further insights into the structure of the *in vitro* reconstituted U1 snRNP, we prepared a sample for electron microscopy. After the size exclusion chromatography, the sample was applied to zonal centrifugation using a glycerol gradient in presence of a fixation agent. After glycerol removal, the particles were imaged by the negative-staining technique. The resulting micrographs revealed a uniform distribution of particles of similar shape that prompted us to prepare cryogenic specimens. A large cryo-EM dataset yielding ∼1.4 million particles was collected. The 2D analysis of the particles revealed the expected projections of U1 snRNP (Figure 2B). However, a high level of conformational heterogeneity was revealed by extensive 3D-classification, which did not allow solving the particle structure at high resolution. The most populated 3D-class was further refined to the resolution of 9.75 Å (Supplementary Figure S3). The cryo-EM map shows the features of U1 snRNP and notably the core of the particles and the two arms that corresponds to the stem loop 1 and stem loop 2. The crystal structure of *in vitro* reconstituted U1 snRNP (18) that is consistent with the structure of U1 snRNP embedded in spliceosome (21) was rigid-body fitted into the cryo-EM map (Figure 2B). The structural model recapitulates the overall shape and dimensions of the RNP and supports the accuracy of our large scale *in vitro* reconstitution protocol.

To observe U1 snRNP by liquid-state NMR spectroscopy, the protein components were expressed in minimal medium partially deuterated (from 70 to 99% D_2_O) allowing uniform ^15^N-labeling and specific ^13^C-labeling on the methyl groups of ILV (70). Unfortunately, U1-C was not expressed in these conditions and could only be expressed at low yield in partially deuterated minimal medium in presence of ^15^N-labeled ammonium chloride. Methyl TROSY NMR fingerprints of the protein components in isolation or in the context of U1 snRNP were recorded (Supplementary Figure S4). Interestingly, when comparing the NMR spectra of isolated components or U1 snRNP, we observed chemical shift changes of the methyl groups. We also produced a sample using only ILV ^13^C-labeled U1 snRNP components except U1-C (Figure 2C). The NMR spectra of U1 snRNP fully labeled or labeled on a single component (U1-A or SmD1-D2) overlay well showing the reproducibility of the sample preparation (Figure 2C). The methyl labeling approach allows us to observe the core of the particle; however, the signals coming from the dynamic part of the particle are clustered in the central region of the spectrum.

### Observation of the U1 snRNP flexible parts

**Figure 3.**
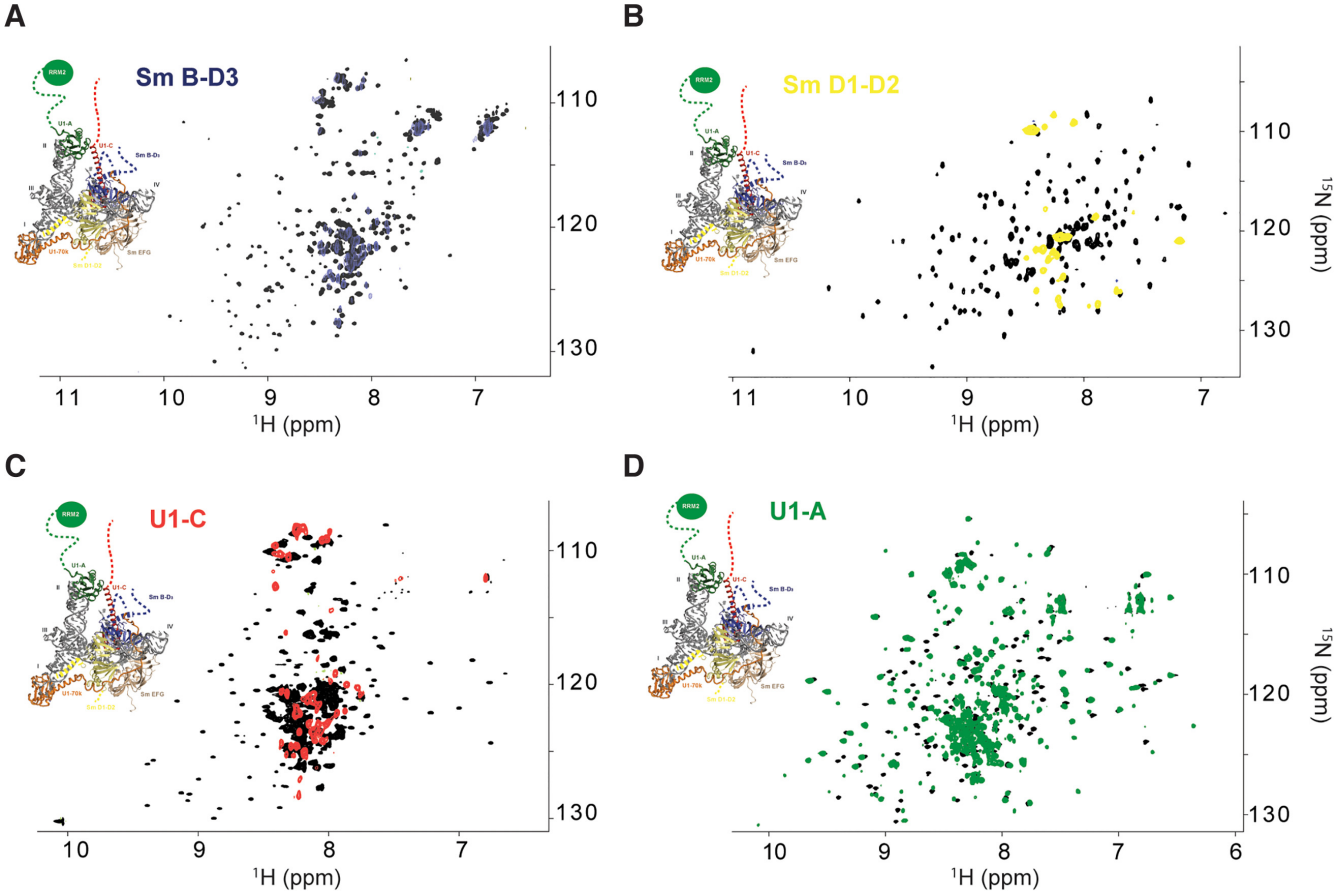
NMR fingerprints of the U1 snRNP tails. (**A**) Overlay of the 2D ^1^H-^15^N TROSY HSQC spectra of Sm B-D_3_ (black) and Sm B-D_3_ embedded in U1 snRNP (blue). (**B**) Overlay of the 2D ^1^H-^15^N TROSY HSQC spectra of Sm D_1_-D_2_ (black) and Sm D_1_-D_2_ embedded in U1 snRNP (yellow). (**C**) Overlay of the 2D ^1^H-^15^N TROSY HSQC spectra of U1-C in complex with unlabeled Sm B-D_3_ (black) and U1-C embedded in U1 snRNP (red). (**D**) Overlay of the 2D ^1^H-^15^N TROSY HSQC spectra of U1-A (black) and U1-A embedded in U1 snRNP (green). On each spectrum, a representation of the 3D structure of U1 snRNP is shown (17).

The *in vitro* assembled U1 snRNP has a molecular weight of ∼220 kDa and tumbles slowly with respect to the NMR timescale making its observation challenging. However, if the U1 snRNP tails reorient faster than the RNP core, their NMR signals should not broaden. To observe the low complexity regions, we first recorded the 2D ^15^N-^1^H HSQC-TROSY fingerprints of isolated U1 snRNP components as well as a 2D {^15^N-^1^H} heteronuclear NOE experiment that reflects backbone flexibility (Supplementary Figure S5). Sm B/B’-D_3_ (Figure 3A), Sm D_1_-D_2_ (Figure 3D) and U1-A (Figure 3D) behaved well in solution and generated highly dispersed NMR spectra, which was not the case for U1-C. As shown by previous structures of U1 snRNP (18,19), U1-C interacts with the Sm core and more specifically with Sm D_3_. In order to disperse the NMR signals of U1-C, we added an equimolar amount of SmB/B’-D_3_ that clearly stabilized U1-C (Figure 3C and Supplementary Figure S6). In all of the NMR spectra of the isolated component of U1 snRNP, a population of signals clusters in the central region of the spectra and corresponds to flexible regions according to ^15^N relaxation experiments (Supplementary Figure S5). The same experiments were recorded after embedding the labeled protein into U1 snRNP. Consequently, only the central parts of the Sm B/B’-D_3_ (Figure 3A), Sm D_1_-D_2_ (Figure 3D) and U1-C (Figure 3C) NMR fingerprints remained observable at almost the same positions as in the free forms. In the context of U1 snRNP, we could observe 74, 38 and 62 remaining NMR signals for Sm B/B’-D_3_, Sm D_1_-D_2_ and U1-C, respectively. Thus, the observed resonances in the context of the U1 snRNP correspond to the flexible segments of Sm B/B’-D_3_, Sm D_1_-D_2_ and U1-C that reorient rapidly compared to the RNP, all the signals belonging to the particle core were broadened beyond detection and vanished. In contrast, for U1-A, all the resonances remained visible once embedded in U1 snRNP (Figure 3D). Drastic changes were observed for the resonances of the RRM1 domain of U1-A, in line with a specific interaction between the RRM1 domain and the tip of the long stem loop 2 (71). Because all the resonances of U1-A remain visible, we propose that both stem loop 2 bulges could induce local flexibility and a faster reorientation rate of the U1-A – stem loop 2 complex that triggers sharper line widths of the signals (71). To conclude, using solution state NMR spectroscopy, the extensions of Sm B/B’-D_3_, Sm D_1_-D_2_, U1-C and U1-A have been observed for the first time in the context of U1 snRNP.

### Probing the structure of U1-A RRM2 in the context of U1 snRNP

**Figure 4.**
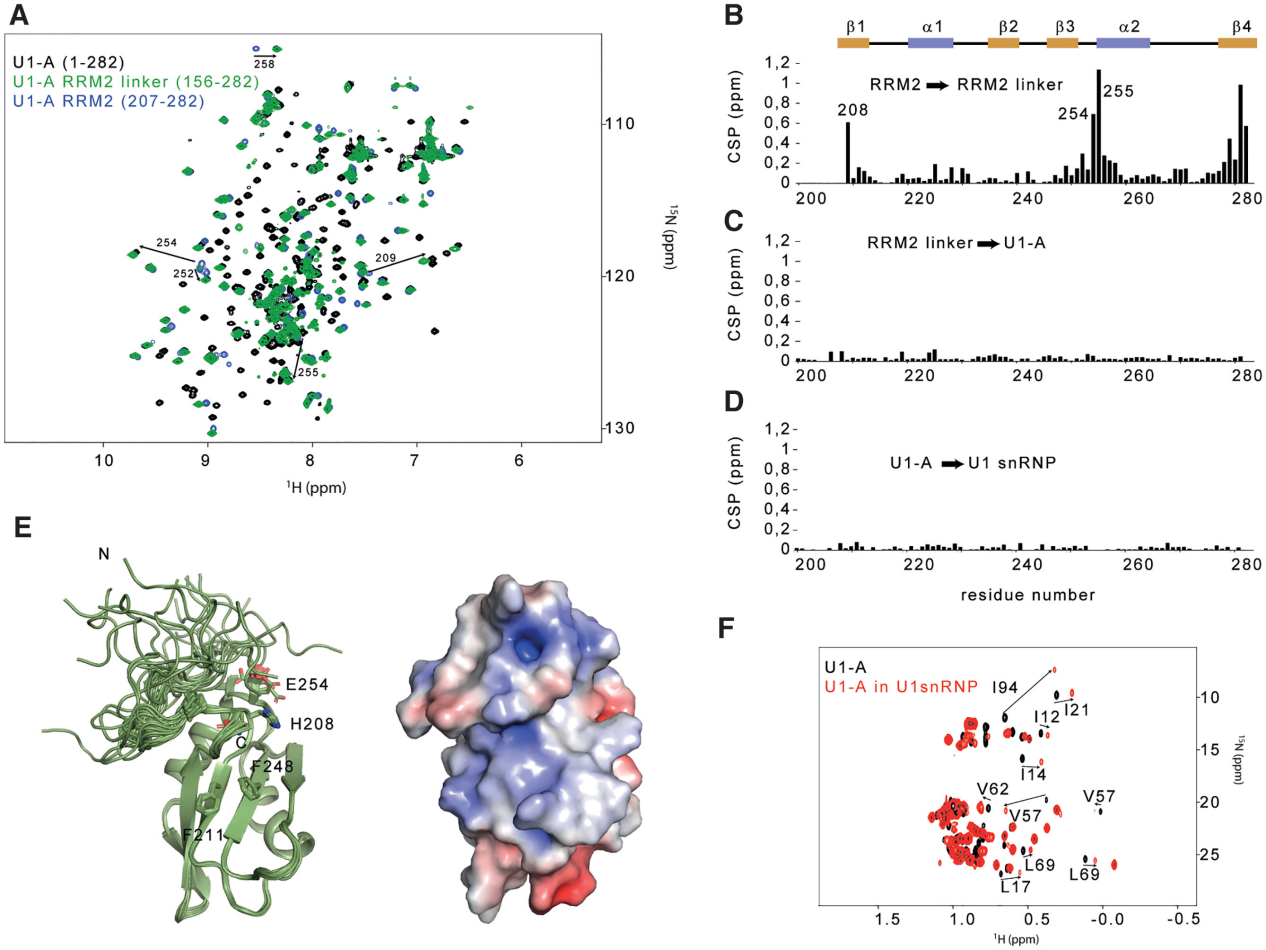
The U1-A RRM2 domain forms an independent structural module in the context of the particle. (**A**) Overlay of the 2D ^1^H-^15^N HSQC spectra of U1-A (1–282, black), U1-A RRM2 linker (156–282, green) and U1-A RRM2 (204–282, blue). (**B**) Plot of the chemical shift perturbations (CSP) in function of the sequence observed between U1-A RRM2 and U1-A RRM2 linker. The CSP cluster at the boundaries of the protein and at the N-terminal tip of helix α2. (**C**) Plot of the CSP as a function of the sequence observed between U1-A RRM2 linker and full-length U1-A. Almost no changes were observed. (**D**) Plot of the CSP as a function of the sequence observed between U1-A and U1-A embedded in U1 snRNP. Almost no changes were observed. (**E**) Ribbon representation of the solution structure of U1-A RRM2 linker (190–282). On the right, the electrostatic surface potential of the lowest energy model is shown. (**F**) Overlay of the 2D ^1^H-^13^C HMQC centred on the methyl group signals of U1-A (black) and U1-A embedded in U1 snRNP (red). All the resonances that shifted are from the RRM1 domain.

The flexible parts of U1 snRNP contain a unique folded domain at the C-terminal extremity of the modular protein U1-A. While the structure and the function of the RRM1 are well documented (71,72), the role of the C-terminal region of U1-A remained poorly understood. We first investigated whether both RNA recognition motifs could interact together. By means of ^15^N relaxation experiments, the longitudinal (R_1_) as well as transverse (R_2_) ^15^N relaxation rates were determined for the full length U1-A protein (1-282), the U1-A RRM2 in isolation (206–282) and the U1-A RRM2 fused to the C-terminal part of the inter-RRM linker (RRM2-linker, 156–282, Figure 4A and Supplementary Figure S7). The ratio between both relaxation rates being directly proportional to the correlation time of the molecule, a strong change between the value determined for the RRM2 in isolation and the full-length U1-A would indicate that both RRMs tumble and interact together in solution. However, the values of *R_1_/R*_2_ were very similar for the RRM2, either isolated or in the context of the full length U1-A. This experimental data demonstrated that both RRM domains tumble independently in solution (Supplementary Figure S7). However, by comparing the 2D ^15^N-^1^H HSQC spectra of RRM2 and RRM2-linker, we observed several chemical shift changes, suggesting that the linker could interact with the RRM2 domain (Figure 4B). To get structural insights into the RRM2 module, we solved the structure of the RRM2-linker protein using 1878 NOE-derived distances. The solution structure revealed that the final part of the linker interacts with the tip of the alpha helix α2 and the beta strand β4, in agreement with the observed chemical shift perturbations (Figure 4F and Table 1). No further changes were observed by comparing the spectra of RRM2-linker and U1-A (Figure 4C). These results showed that the remaining part of the linker does not contact RRM2. To probe the structure of U1-A RRM2 in the context of U1 snRNP, the chemical shifts of the isolated RRM2 were compared with the ones observed in U1 snRNP and revealed almost no change (Figure 4D), suggesting that the C-terminal part of U1-A (156–282) is independent from the core of the particle. In addition, by comparing the methyl group fingerprints of U1-A in solution or in the context of U1 snRNP, significant changes were observed for the RRM1 signals while the ones of RRM2 remained unaffected (Figure 4F). Overall, our results demonstrate that the RRM2 domain of U1-A interacts with the initial part of the linker to form an independent domain and its structure remains unaffected in the context of U1 snRNP.

**Table 1.** NMR refinement statistics

	U1-A RRM2 linker
**NMR distances and dihedral constraints**	
Distance constraints	
Total NOE	1840
Intra-residue	387
Inter-residue	1453
Sequential (|*i* – *j*| = 1)	444
Medium-range (1<|*i* – *j*| < 4)	308
Long-range (|*i* – *j*| > 5)	671
Hydrogen bonds	30
Total dihedral angle restraints	
*ϕ*	72
*ψ*	72
**Structure statistics**	
Violations (mean ± s.d.)	
Distance constraints (Å) > 0.4 Å	0.30 ± 0.50
Dihedral angle constraints (°) > 5°	5.00 ± 1.40
Max. dihedral angle violation (°)	7.49 ± 2.67
Max. distance constraint violation (Å)	0.37 ± 0.13
Deviations from idealized geometry	
Bond lengths (Å)	0.0035 ± 0.0012
Bond angles (°)	1.443 ± 0.481
Ramachandran plot statistics	
Residue in most favored regions (%)	84.4
Residue in additionally allowed regions (%)	15.6
Residue in generously allowed regions (%)	0.0
Residue in disallowed regions (%)	0.0
Average pairwise r.m.s. deviation (Å)	
Heavy	0.12 ± 0.05^a^
Backbone	0.50 ± 0.06^a^

^a^Pairwise RMSD calculated among the 20 NMR structures using residue range 208–240, 244–282.

### Probing the direct interaction between U1 snRNP and the splicing factor PTBP1 in solution

**Figure 5.**
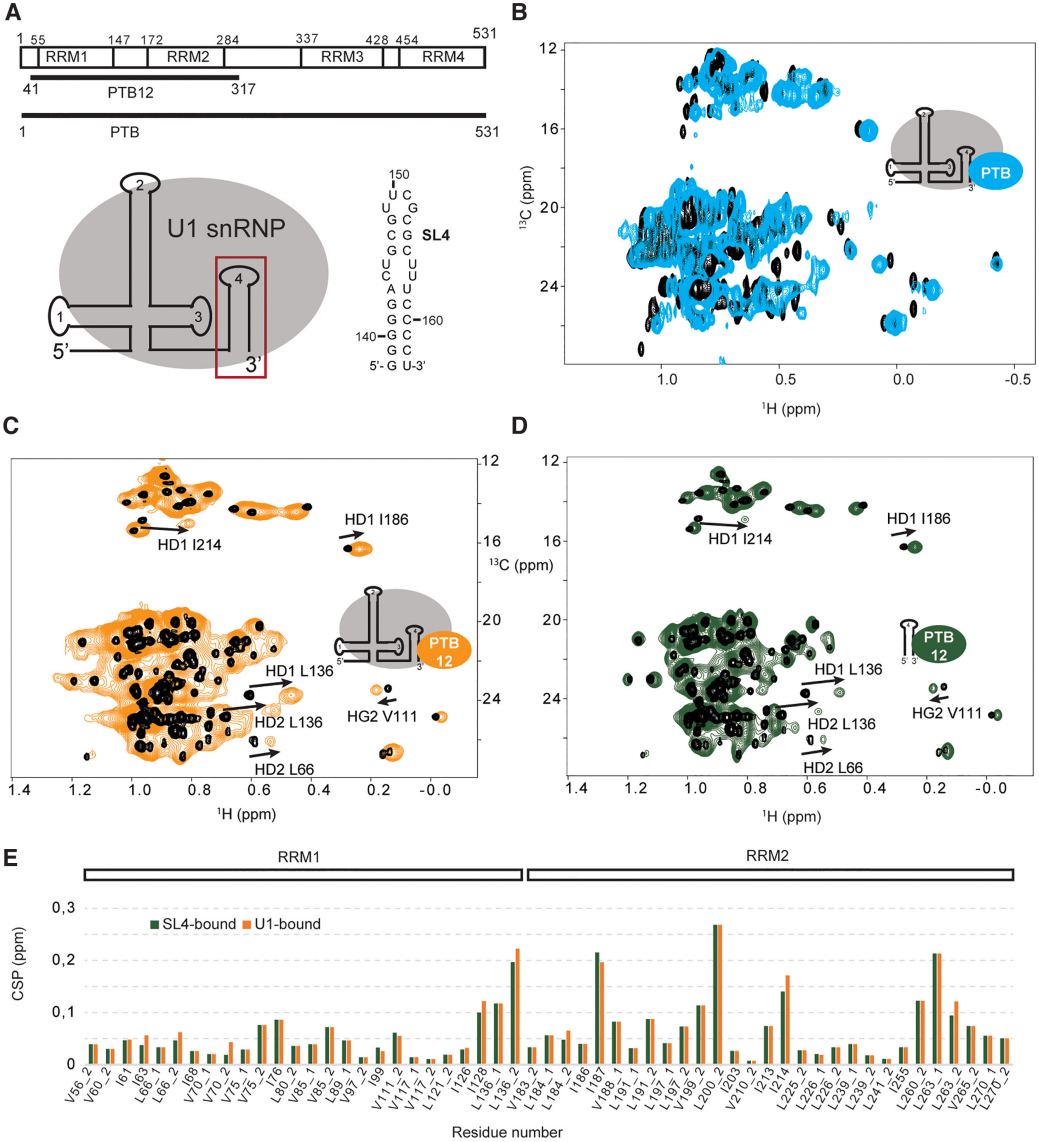
Interaction between U1 snRNP and the splicing factor PTBP1 monitored by NMR spectroscopy. (**A**) Schematic representation of the PTBP1 constructs and of the RNA stem loop sequence. (**B**) Overlay of the 2D ^1^H-^13^C HMQC spectra of the free PTBP1 protein (black) and in complex with U1 snRNP (cyan). (**C**) Overlay of the 2D ^1^H-^13^C HMQC spectra of the free PTBP1 N-terminal half (PTB12, black) and in complex with U1 snRNP (orange). (**D**) Overlay of the 2D ^1^H-^13^C HMQC spectra of the free PTBP1 N-terminal half (PTB12, black) and in complex with U1 stem loop 4 (green). (**E**) Plot showing the chemical shift perturbations (CSP) of the methyl groups of ILV of PTBP1 N-terminal observed upon addition of U1 snRNP (orange) or U1 snRNA SL4 (green). Methyl groups are labeled according to the residue number and _1 or _2 stands for HD1/CD1 and HD2/CD2 in the case of leucine or HG1/CG1 and HG2/CG2 in the case of valine.

The interactions controlling alternative splicing are transient and labile and we thought that solution NMR spectroscopy might be an appropriate method to get structural insights into the weak interactions between the splicing factors and U1 snRNP. The splicing factor PTBP1 was previously proposed to directly interact with U1 snRNA terminal stem loop 4 in nuclear extracts. The individual N-terminal RRM1 and RRM2 were shown to bind stem loop 4 with a better affinity than a short UCUCU single-stranded RNA oligonucleotide (34). To verify that this interaction holds in the context of the particle, we monitored the binding of PTBP1 to U1 snRNP in solution (Figure 5A and B). Upon addition of U1 snRNP, the PTBP1 ILV methyl groups of all the four RRMs experienced chemical shift changes and line broadening, in agreement with the formation of several complexes and conformational exchange. To simplify the system, we repeated a similar experiment by replacing the full-length PTBP1 by the N-terminal half of the protein containing both RRM1 and 2 (PTB12), previously proposed as the main U1 snRNA stem loop 4 binders (34). Upon formation of the complex, both RRM domains experienced methyl group chemical shift changes showing that both domains contact the particles (Figure 5C). Similar changes were reproduced when the particle was replaced by the isolated stem loop 4 (Figure 5D and E). By studying the interaction between PTBP1 and U1 snRNP using NMR spectroscopy, we confirmed that PTBP1 interacts with the terminal stem loop 4 of U1 snRNP through its N-terminal half and does not contact any protein from U1 snRNP. Since the chemical shifts of the RRM1 and 2 are similar when the protein was titrated by the U1 snRNP particle or the terminal stem loop 4, we provided an experimental evidence that PTBP1 contacts only the RNA component of U1 snRNP *in vitro* and more precisely stem loop 4.

## DISCUSSION

The spliceosome is a dynamic molecular machine, known for its stepwise assembly and its high content of intrinsically disordered regions. While the particle cores were characterized at the atomic level (2), the low complexity regions, which are also functionally relevant, remained invisible. In this study, we reconstituted U1 snRNP *in vitro* using mostly full-length proteins produced in bacteria and probed its shape and structure. Using electron microscopy, we obtained a low resolution structure that validated the success of the reconstitution. By producing each component individually, our approach offers all the possible isotope labeling schemes. Here, we developed an NMR spectroscopy-based approach to observe the U1 snRNP tails in solution as it has already been done for other particles of similar size or even bigger (73–76).

In the context of U1 snRNP, the NMR signals of the Sm tails, the C-terminal parts of U1-C and U1-A remained visible due to their flexibility, suggesting that the U1 snRNP tails reorient faster than the particle core in solution. These intrinsically disordered regions protrude from the particle core, extend the solvent exposed surface of U1 snRNP and could act as additional protein–protein interaction surfaces involved in the splicing mechanism or its regulation. In line with our results, the cryo-EM structure of U1 snRNP from *Saccharomyces cerevisiae* showed that the flexible tail of U1-C has the capacity to fold upon protein partner recruitment and suggested that it could be also the case in higher eukaryotes (67). The resonance assignment of the U1 snRNP components and the measurement of orientational restraints could help modeling the flexible tails using recently developed modeling approaches (77,78). More surprisingly, the amide signals of the entire U1-A remained observable in the context of the RNP, indicating a faster tumbling rate of the U1-A-stem loop 2 complex compared to the rest of the particle. This dynamic feature of the stem loop 2 is in agreement with previous observation showing that this part of the particle prevents the formation of well-ordered crystals (18). However, it is not yet known if the flexibility of stem loop 2 is functionally relevant. The U1 snRNP tails contain a single globular domain at the C-terminus of U1-A (RRM2). Here, we probed its structure in the context of U1 snRNP and showed that U1-A RRM2 tumbles independently from the particle core. However, the function of this domain still remains elusive. In the nucleus, transcription and RNA processing cooperate physically and cluster in restricted area phase separated from the rest of the nucleus (79,80). The *in vitro* reconstituted U1 snRNP that lacked the RS domain of U1–70K did not induce the formation of liquid droplets (at micromolar concentrations). Since we have shown recently that U1 snRNP strongly associates with FUS in the nucleus (33), it might be possible that the particle associates with FUS or other proteins well known to drive liquid–liquid phase separation to achieve subnuclear compartmentation. Future biophysical studies should address this question and in particular the role of the flexible tails of U1 snRNP in this process.

By producing U1 snRNP for solution state NMR spectroscopy, we opened the door to the study of direct interactions between splicing factors and U1 snRNP that drive alternative splicing. Here, we could confirm that the splicing modulator PTBP1 directly contacts U1 snRNP via the RNA component of the particle. In solution, the PTBP1 N-terminal half experienced similar chemical shift changes when titrated by U1 snRNP or U1 snRNA stem loop 4. This result clearly supports that PTBP1 contacts the stem loop 4 when it interacts with the first particle of the spliceosome. The stem loop 4 also establishes direct contacts to the U2 snRNP component SF3A1 during pre-spliceosomal A complex formation (36). Are PTBP1 and SF3A1 competing for the binding of stem loop 4 or could they be accommodated simultaneously on the same U1 snRNP particle? PTBP1 was proposed to bind the pyrimidine rich internal loop of SL4 (34) and the ubiquitin-like domain of SF3A1 seems to target the double stranded region of the stem loop (36). With both RRM1 and RRM2 of PTBP1 binding to SL4, these interactions are likely to be mutually exclusive. Deciphering the atomic details of the interactions between PTBP1, SF3A1 and U1 snRNP stem loop 4 might provide insights into this potential mechanism of splicing regulation. We have also shown that the splicing factor FUS interacts with the stem loop 3 of U1 snRNP using a similar NMR spectroscopy approach, these results were confirmed *in vivo* using CLIP experiments (33). The approach developed here allows a precise mapping of the interaction surface between U1 snRNP and any splicing factors. In addition, we could already show that the binding of small molecule ligand to the *in vitro* reconstituted U1 snRNP can be monitored by NMR spectroscopy (17). In this case, we took advantage of the presence of a fluorine on the small molecule ligand to access its association with U1 snRNP/5′-ss complexes. Probing the direct interactions between U1 snRNP and natural or artificial splicing factors can be achieved with the NMR spectroscopy approach described in this manuscript. It might also represent an interesting way to screen for small molecule therapeutics that modulate the communication between U1 snRNP, splicing factors and/or the pre-mRNA target.

## DATA AVAILABILITY

NMR derived atomic coordinates of the U1-A RRM2-linker have been deposited in the Protein Data Bank (doi:10.2210/pdb7AEP/pdb) under the following accession code 7AEP. The NMR spectroscopy chemical shifts and restraints have been deposited in the BioMagResBank (doi:10.13018/BMR34560) under the following accession codes 34560.

## Supplementary Material

gkab135_Supplemental_File
