# Supplementary material for: An in vitro reconstituted U1 snRNP allows the study of the disordered regions of the particle and the interactions with proteins and ligands
Source: Nucleic Acids Res. 2021 Mar 2;49(11):e63. doi: 10.1093/nar/gkab135 (PMC8216277; doi:10.1093/nar/gkab135)
Supplement: gkab135_Supplemental_File [file gkab135_supplemental_file.pdf]

Supplementary data

An in-vitro reconstituted U1 snRNP allows the study of the disordered regions of the particle and the interactions with proteins and ligands

Sébastien Campagne[1,*], Tebbe de Vries[1], Florian Malard[1], Pavel Afanasyev[2], Georg Dorn[1], Emil Dedic[1], Joachim Kohlbrecher[3], Daniel Boehringer[2], Antoine Cléry[1] and Frédéric H-T. Allain[1,*]

[1]Institute of Biochemistry, Department of Biology, Swiss Federal Institute of Technology, Zurich, Switzerland.

[2]Cryo-EM Knowledge Hub (CEMK), Swiss Federal Institute of Technology, Zurich, Switzerland.

[3]Paul Scherrer Institute, SINQ facility, Villigen, Switzerland

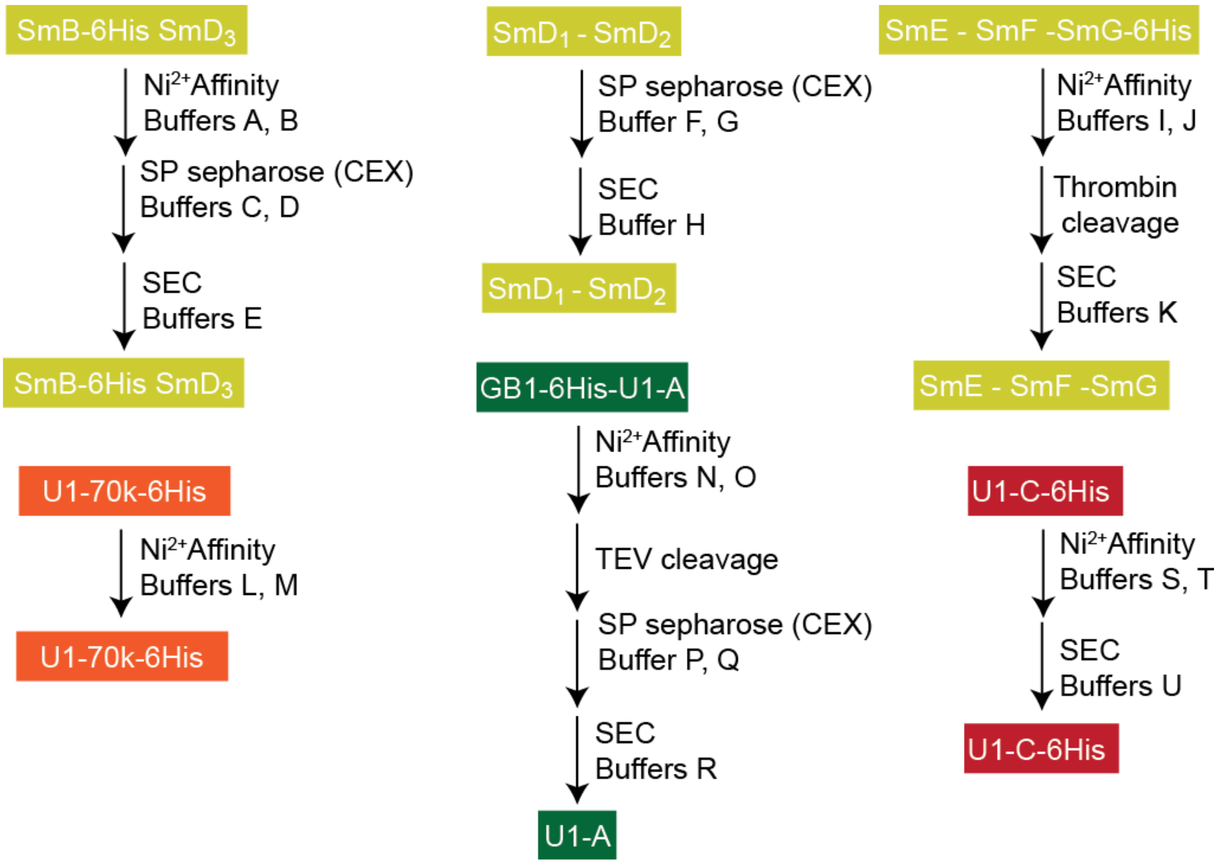

Supplementary Figure 1 – Purification scheme of the U1 snRNP protein components. Buffer compositions and workflow are described in the supplementary method section.

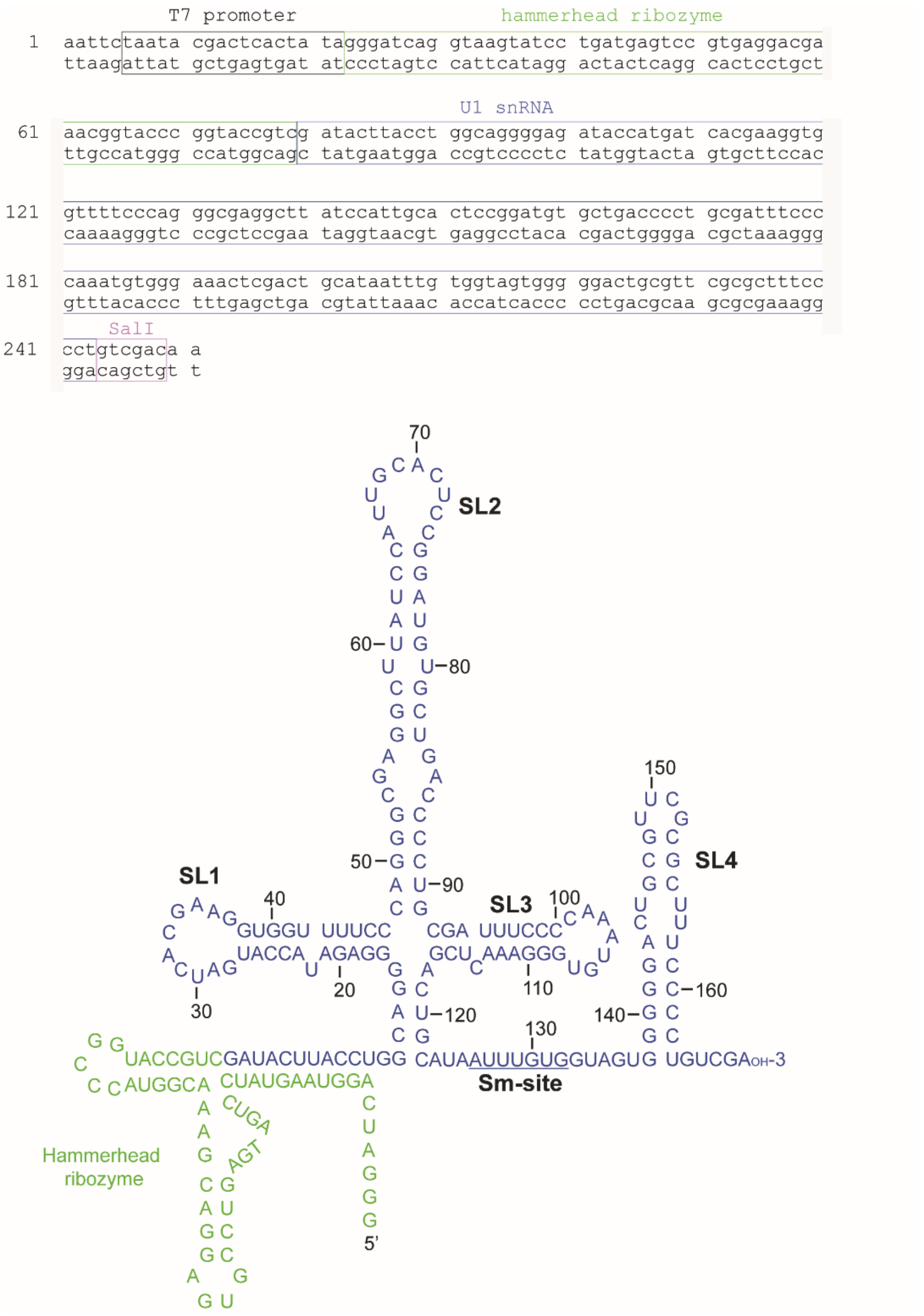

Supplementary Figure 2 – Template for the *in vitro* transcription of the U1 snRNA.

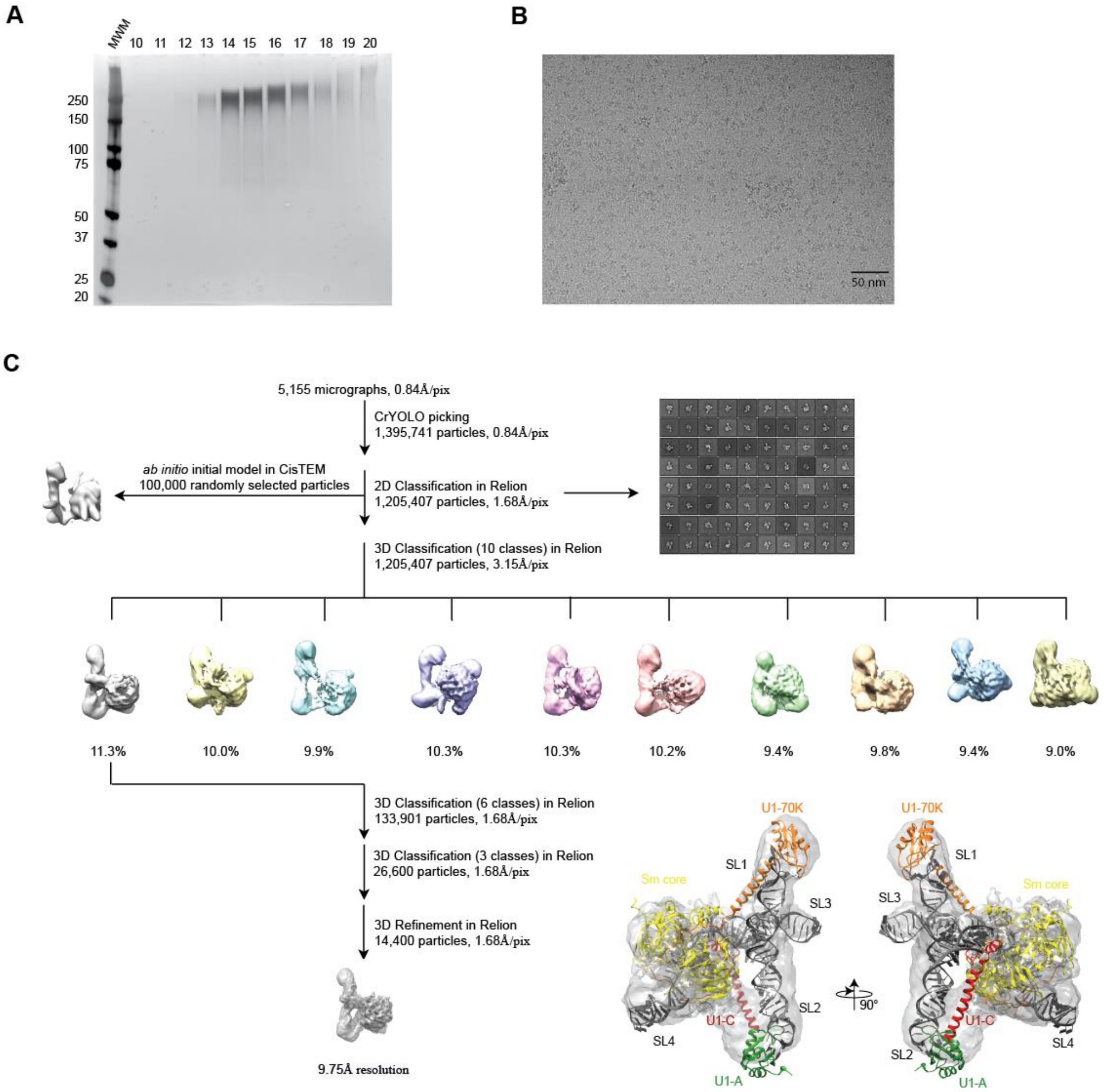

Supplementary Figure 3 – Single-particle cryo-EM of the *in vitro* reconstituted U1 snRNP. (A) SDS PAGE gel showing the fractionation of the glycerol gradient 10-30% performed in presence of glutaraldehyde. The gel was stained with silver nitrate. (B) Cryo-EM micrograph of the *in vitro* reconstituted U1 snRNP. (C) Scheme showing the single-particle analysis workflow.

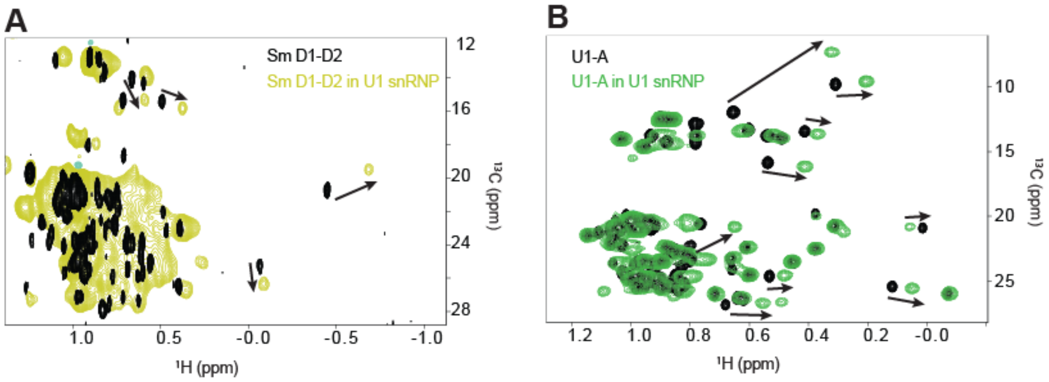

Supplementary Figure 4 – Monitoring the formation of U1 snRNP using methyl group signals. (A) Comparison of the 2D ^{1}H ^{13}C HMQC spectra of Sm D_1-D_2 in isolation (black) or embedded into U1 snRNP (yellow). (B) Comparison of the 2D ^{1}H ^{13}C HMQC spectra of U1-A in isolation (black) or embedded into U1 snRNP (green). All the methyl groups that experienced chemical shift perturbations are from the RRM1 domain while the methyl groups of the RRM2 domain remain unchanged upon formation of the particle.

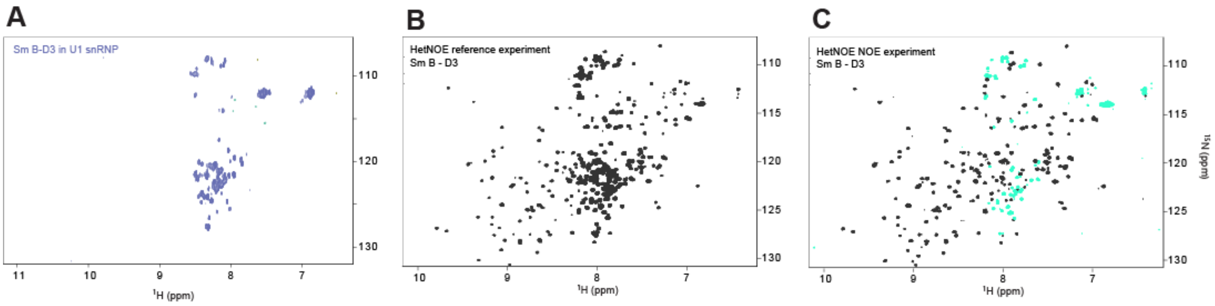

Supplementary Figure 5 – The C-terminal tails of SmB-D_3 remain flexible in U1 snRNP. (A) 2D ^{15}N-^{1}H TROSY HSQC spectrum of SmB-D_3 embedded in U1 snRNP (blue). In U1 snRNP, only the central part of the spectrum was observed. (B-C) Determination of the {^{15}N-^{1}H} heteronuclear NOE value of the isolated Sm B-D_3. The reference spectra and the spectra recorded after saturation of the amide protons are depicted in B and C, respectively. The intensity ratio between B and C determines the value of the {^{15}N-^{1}H} heteronuclear NOE. When the value is close to 1, the residue is considered as rigid while a value lower than 0.25 supports flexibility. All the residues that show low values of {^{15}N-^{1}H} heteronuclear NOE remain visible in U1 snRNP. Thus, the flexible parts of SmB-D_3 remain visible in U1 snRNP.

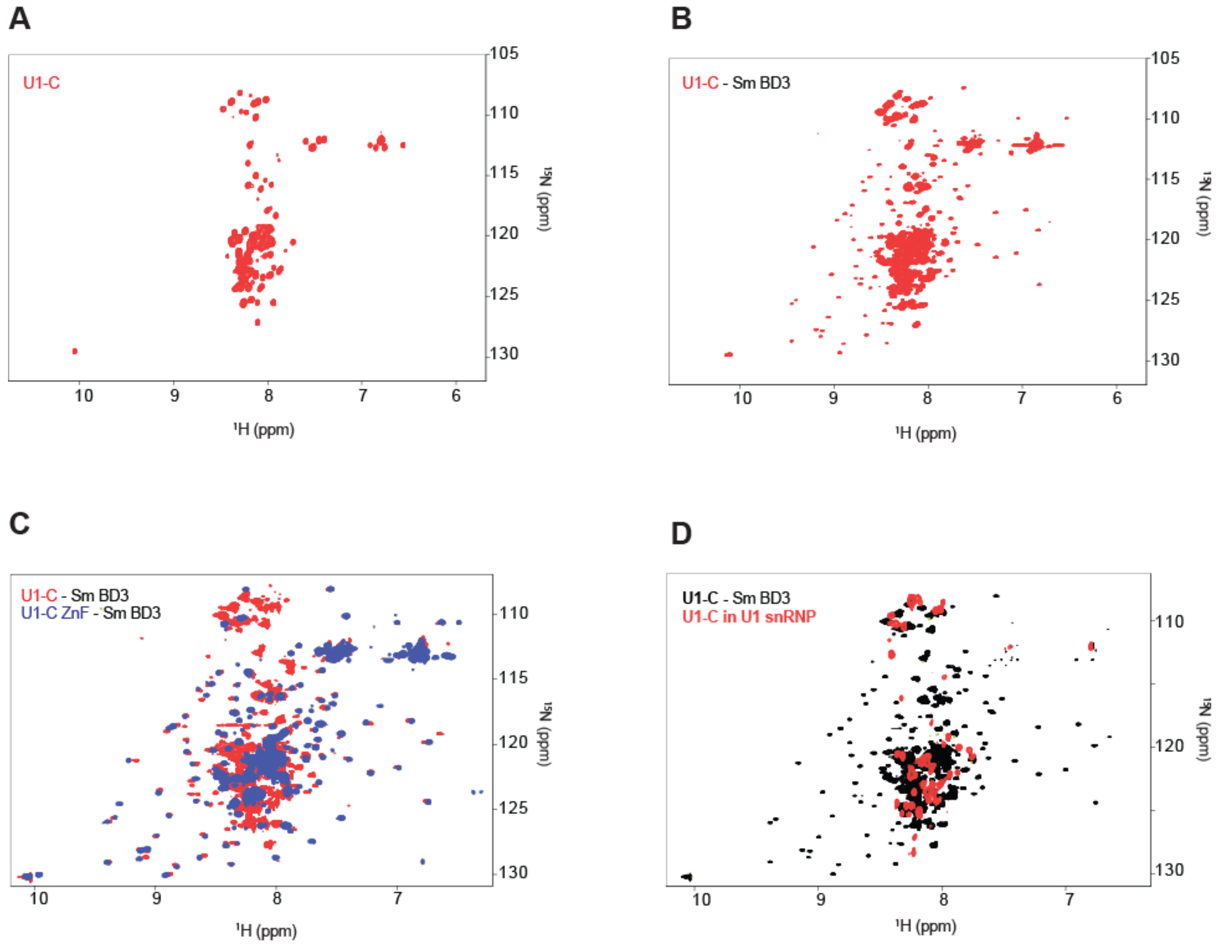

Supplementary Figure 6 – U1-C folds upon binding to SmB-D$_3$. (A) 2D ^{15}N-^{1}H HSQC spectrum of U1-C. (B) 2D ^{15}N-^{1}H HSQC spectrum of U1-C in presence of one equimolar amount of SmB-D$_3$. Upon addition of SmB-D$_3$, the resonances of U1-C become dispersed. (C) Overlay of the 2D ^{15}N-^{1}H HSQC spectra of U1-C-SmB-D$_3$ (red) and of the U1-C zinc finger domain (1-61) in complex with SmB-D$_3$ (blue). In B and C, SmB-D$_3$ is not labelled and therefore invisible on the 2D ^{15}N-^{1}H HSQC spectra. The blue spectrum corresponds to the folded of U1-C and it overlaps very well with the spectra of full length U1-C. The additional resonances observed on the U1-C spectrum (red) correspond to the C-terminal tail of U1-C and the signals are in the centre of the spectrum, in the random coiled region. (D) Overlay of the 2D ^{15}N-^{1}H HSQC TROSY of U1-C in complex with SmB-D$_3$ (black) or in U1 snRNP (red). The observed NMR signals in the context of U1 snRNP correspond to the C-terminal tail while the signals from the zinc finger domain, that take part of the core of the particle, are broadened and bleached from the NMR spectra.

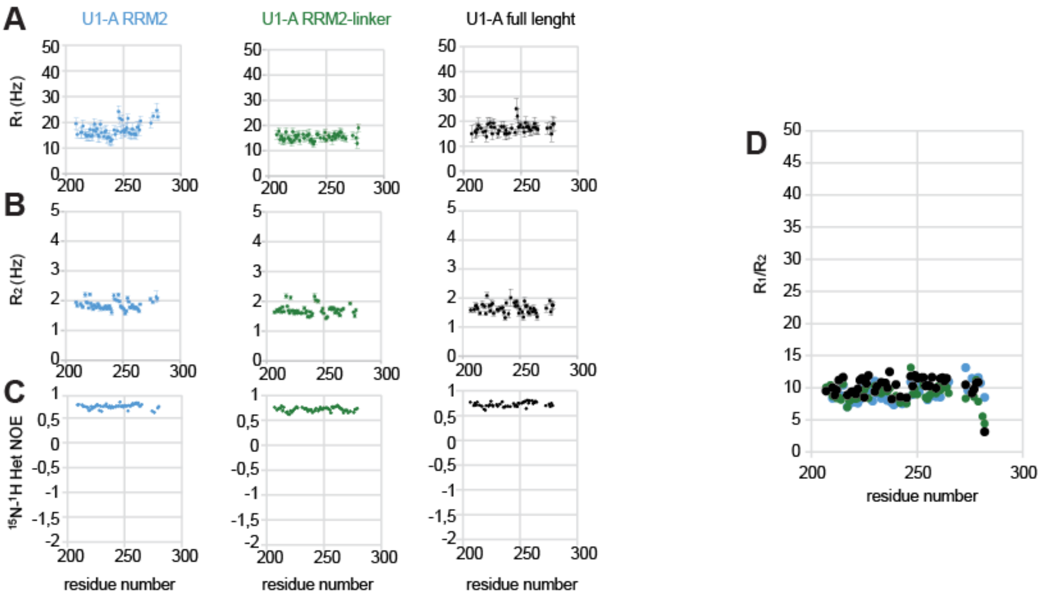

Supplementary Figure 7 – Both RRM domains of U1-A tumble independently in solution. (A) Plots of the ^{15}N longitudinal relaxation rates (R_1) of the RRM2 domain of U1-A as a function of the residue number. These measurements have been performed with the isolated RRM2 domain (blue), RRM2 fused to the C-terminal part of the linker (green) and U1-A full length (black). (B) Plots of the ^{15}N transverse relaxation rates (R_2) of the RRM2 domain of U1-A as a function of the residue number. (C) Plots of the values of the {^{15}N-^{1}H} heteronuclear NOE of the RRM2 domain of U1-A as a function of the residue number. (D) Plot of the ratio R_1/R_2 in function of the sequence of RRM2. The values determined for the isolated RRM2 (blue), RRM2 fused to the C-terminal part of the linker (green) or U1-A (black) are very similar. This ratio being proportional to the correlation time of the molecule in solution, we concluded that the RRM2 domain tumbles independently in the context of the full-length protein.

Supplementary methods

Cloning and expression of U1 snRNP protein components. The Sm protein ORFs were combined into polycistronic genes and inserted into pET26bII to express the following combinations: Sm B-D_3, Sm D_1-D_2 and Sm E-F-G. The expression of Sm B/B'-D_3, Sm D_1-D_2 and Sm E-F-G have been performed in *Escherichia coli* BL21 DE3 at 20°C, 25°C and 37°C during 12h, 12h and 24h, respectively. The full length U1-A (1-282), U1-A RRM1 (1-127), U1-A RRM2 (207-282) and U1-A RRM2-linker (156-282) open reading frames (ORF) were cloned into pET26bII in fusion with a hexa-histidine tag cleavable by thrombin. The U1-C (1-159) and U1-70K (1-216) ORFs were cloned in fusion with a C-terminal hexa-histidine tag using the pET28a plasmid. The U1-C zinc finger was cloned in fusion with a GST tag cleavable by thrombin using pGEX-4T3. U1-A, U1-C and U1-70K were expressed in *Escherichia coli* BL21 DE3 at 37°C, 25°C and 20°C, respectively. Isotopically labelled proteins were expressed in M9 medium complemented with either 1 g/L of $^{15}NH_4Cl$ (for ^{15}N labelling), 2 g/L of ^{13}C glucose (for ^{13}C labelling), 100 mg/L of alpha-ketobutyric acid (methyl-^{13}C, 99%; 3,3-D2, 98%, Cambridge Isotope Laboratory) and 60 mg/L of alpha-ketoisovaleric acid ($^{13}C5$, 98%; 3-D1, 98%, Cambridge Isotope Laboratory) for ^{13}C labelling of ILV methyl groups. In order to partially deuterated the proteins, the M9 medium was prepared with D_2O instead of H_2O.

Purification of the U1 snRNP protein components. Sm B/B'-D_3 was solubilized in buffer A (Hepes 20 mM pH 7.8, NaCl 1 M, Urea 2 M, β-mercapto-ethanol 2.8mM) in presence of 10ug/ml of DNAseI and lysozyme. The cells were lysed using a microfluidizer using 5 consecutive cycles at 15,000 psi. The cell lysate was clarified by centrifugation (30,000g at 4°C during 45 minutes) and loaded at 0.5 ml/min on a 5 ml HisTrap column (GE Healthcare) at 4°C. The column was washed with buffer A (100 ml) and 10% B (50 ml) and eluted with buffer B (Hepes 20 mM pH 7.8, NaCl 1 M, imidazole 500 mM, β -mercapto-ethanol 2.8 mM). The heterodimer Sm B-D3 was cleaved by thrombin (Sigma) and dialyzed in buffer C (sodium

phosphate 10 mM pH6.0, DTT 5mM). The sample was then loaded on the HiTrap SP 5ml column, washed with buffer C and eluted with buffer D (sodium phosphate 10 mM pH6.0, 2 M NaCl, DTT 5mM). Finally, the protein was dialyzed with buffer E (sodium phosphate 10 mM pH6.0, DTT 5mM) and loaded on a size exclusion column (SEC) previously equilibrated with buffer E. The protein was concentrated to 100 µM and stored at -80°C.

Sm D_1-D_2 was solubilized in buffer F (Hepes 20 mM pH 7.4, NaCl 0.5 M, Urea 0.5 M, DTT 10 mM, EDTA 1mM). The cells were lysed using a microfluidizer using 5 consecutive cycles at 15,000 psi. The cell lysate was clarified by centrifugation (30,000g at 4°C during 45 minutes) and loaded at 2 ml/min on a 5 ml HiTrap SP column (GE Healthcare) at 4°C, washed with buffer F and eluted with buffer G (Hepes 20 mM pH 7.4, NaCl 2 M, DTT 10 mM, EDTA 1mM). Sm D1-D2 was further purified by SEC in buffer H (sodium-phosphate buffer 10 mM pH 6.8, NaCl 50 mM, DTT 5 mM). The protein was concentrated to 100 µM and stored at -80°C.

Sm E-F-G was solubilized in buffer I (Hepes 20 mM pH 7.5, NaCl 0.5 M, Urea 0.5 M, imidazole 10 mM, β -mercapto-ethanol 2.8 mM) in presence of 10ug/ml of DNAseI and lysozyme. The cells were lysed using a microfluidizer using 5 consecutive cycles at 15,000 psi. The cell lysate was clarified by centrifugation (30,000g at 4°C during 45 minutes) and loaded at 2 ml/min on a 5 ml HisTrap column (GE Healthcare) at room temperature, washed with buffer H and eluted with buffer J (Hepes 20 mM pH 7.5, NaCl 0.5 M, imidazole 500 mM, β -mercapto-ethanol 2.8 mM). The heterotrimer Sm E-F-G was cleaved by thrombin (Sigma), dialyzed in buffer K and further purified by SEC in buffer K (Hepes 20 mM pH 7.5, NaCl 0.5 M, DTT 5 mM). The protein was concentrated to 100 µM and stored at -80°C.

U1-70K (1-216) was solubilized in buffer L (Hepes 20 mM pH 7.5, NaCl 1 M, Urea 0.5 M, imidazole 10 mM, β -mercapto-ethanol 2.8 mM) in presence of 10ug/ml of DNAseI and lysozyme. The cells were lysed using a microfluidizer using 5 consecutive cycles at 15,000 psi.

The cell lysate was clarified by centrifugation (30,000g at 4°C during 45 minutes) and loaded at 2 ml/min on a 5 ml HisTrap column (GE Healthcare) at room temperature previously equilibrated with buffer K. The column was extensively washed with buffer K and eluted with a linear gradient of buffer M (Hepes 20 mM pH 7.5, NaCl 0.5 M, imidazole 300 mM, β-mercapto-ethanol 2.8 mM). Fractions enriched for U1-70k (~10 μM) were flash frozen in liquid nitrogen and stored at -80°C.

U1-A was solubilized in buffer N (Hepes 20 mM pH 7.5, NaCl 0.5 M, Urea 0.5 M) in presence of 10ug/ml of DNAseI and lysozyme. The cells were lysed using a microfluidizer using 5 consecutive cycles at 15,000 psi. The cell lysate was clarified by centrifugation (30,000g at 4°C during 45 minutes) and loaded at 2 ml/min on a 5 ml HisTrap column (GE Healthcare) at room temperature, washed with buffer N and eluted with buffer O (Hepes 20 mM pH 7.5, NaCl 0.5 M, imidazole 300 mM). The protein was cleaved by TEV protease at room temperature and diluted 5 times to reduce the NaCl concentration to 100 mM. The cleavage reaction was loaded on a 5 ml HiTrap SP column (GE Healthcare) at room temperature previously equilibrated with buffer P (Hepes 20 mM pH 7.5, NaCl 0.1 M), washed with buffer P and U1-A was eluted with buffer Q (Hepes 20 mM pH 7.5, NaCl 2 M), concentrated and further purified by SEC in buffer R (sodium-phosphate buffer 10 mM pH 6.8, NaCl 50 mM, EDTA 0.5 mM, DTT 5 mM). The protein was concentrated to 100 µM and stored at -80°C.

U1-C was solubilized in buffer S (Hepes 10 mM pH 7.8, NaCl 0.1 M, Urea 0.5 M, β-mercapto-ethanol 2.8 mM) in presence of 10ug/ml of DNAseI and lysozyme. The cells were lysed using a microfluidizer using 5 consecutive cycles at 15,000 psi. The cell lysate was clarified by centrifugation (30,000g at 4°C during 45 minutes) and loaded on a 5 ml HisTrap column (GE Healthcare) at 4°C. The column was washed with buffer S, 10% buffer T (Hepes 10 mM pH 7.8, NaCl 0.1 M, Urea 0.5 M, Imidazole 250 mM, β-mercapto-ethanol 2.8 mM) and U1-C was eluted with 100% buffer T. The protein was concentrated at 4°C and further purified by SEC in

buffer U (sodium-phosphate buffer 10 mM pH 6.8, NaCl 50 mM, DTT 5 mM). Elution fractions containing ~10 µM of U1-C were flash frozen in liquid nitrogen and stored at -80°C.

Cloning of the plasmid allowing the transcription of U1 snRNA and its derivative. A plasmid (pUC19-U1 snRNA) containing a T7 promoter followed a Hammerhead ribozyme fused to the U1 snRNA was ordered (Genscript). The plasmid was linearized by SalI. The construct is described in Supplementary Figure 2.
